# Applicability of drinking water treatment residue for lake restoration in relation to metal/metalloid risk assessment

**DOI:** 10.1038/srep38638

**Published:** 2016-12-08

**Authors:** Nannan Yuan, Changhui Wang, Yuansheng Pei, Helong Jiang

**Affiliations:** 1 State Key Laboratory of Lake Science and Environment, Nanjing Institute of Geography and Limnology, Chinese Academy of Sciences, Nanjing 210008, China; 2 The Key Laboratory of Water and Sediment Sciences, Ministry of Education, School of Environment, Beijing Normal University, Beijing 100875, P. R. China

## Abstract

Drinking water treatment residue (DWTR), a byproduct generated during potable water production, exhibits a high potential for recycling to control eutrophication. However, this beneficial recycling is hampered by unclear metal/metalloid pollution risks related to DWTR. In this study, the pollution risks of Al, As, Ba, Be, Cd, Co, Cr, Cu, Fe, Mn, Mo, Ni, Pb, and Zn due to DWTR application were first evaluated for lake water based on human health risk assessment models and comparison of regulatory standards. The risks of DWTR were also evaluated for sediments on the basis of toxicity characteristics leaching procedure and fractionation in relation to risk assessment code. Variations in the biological behaviors of metal/metalloid in sediments caused by DWTR were assessed using *Chironomus plumosus* larvae and *Hydrilla verticillata*. Kinetic luminescent bacteria test (using *Aliivibrio fischeri*) was conducted to analyze the possibility of acute and chronic detrimental effects of sediment with DWTR application. According to the obtained results, we identify a potential undesirable effect of DWTR related to Fe and Mn (typically under anaerobic conditions); roughly present a dosage threshold calculation model; and recommend a procedure for DWTR prescreening to ensure safe application. Overall, managed DWTR application is necessary for successful eutrophication control.

Water management faces a global call to control excessive phosphorous (P) for lake restoration^1^. Internal P released from sediment has been considered a major source of excess P in lake water^2^. Typically, an *in situ* geo-engineering technique referred to as chemical treatment has been shown to be an effective method for internal P pollution control^3^,^4^. The technique is to reduce the mobility of P in lake sediment by dosing reactive materials. Satisfactory results have thus far been achieved by using various commercial materials, such as aluminum (Al) salts^5^, iron (Fe) salts^6^, and La-modified bentonite clay (Phoslock^®^)^7^. However, to obtain environmental benefits and attain economic viability, low-cost industrial byproducts^8^ and naturally occurring or modified mineral complexes^9^,^10^ have also been tested for eutrophication control.

Drinking water treatment residue (DWTR), an inevitable byproduct generated during potable water production, has drawn increasing interest for environmental recycling. Recycling approaches can generally be classified into flocculant recovery^11^, soil improvement^12^, and environmental remediation^13^,^14^. Traditionally, DWTR is primarily composed of Al and Fe hydroxides because of flocculant utilization in water treatment. This composition leads to its strong adsorption capability for many contaminants, such as metals/metalloids and organic compounds^15^,^16^. This composition is also typically used for P adsorption^17^. Many researchers have also reused DWTR for P control in the environment. For example, DWTR has been reused as soil amendment for off-site P pollution control^18^ and as substrate in constructed wetlands to remove excessive P from wastewater^19^.

Recently, reuse of DWTR as a reactive material has been attempted for *in situ* chemical treatment to control eutrophication^20^,^21^. Immobilized P in lake sediment caused by DWTR has been shown to exhibit high stability under varied conditions, e.g., pH (in the range of 5–9), dissolved oxygen, ion strength, organic matter, and silicate^22^. Aging also exerts a limited effect on the P immobilization capability of DWTR in lake water because of the inhibition of Al and Fe crystallization caused by ligands, e.g., organic matter, phosphate, and silicate contained in DWTR^23^. Moreover, DWTR addition could induce conditions that are beneficial to anaerobic ammonium oxidation and nitrification in lake sediments^24^,^25^. Successful DWTR application can lead to another win-win situation for environmental remediation.

Nonetheless, DWTR is a sink for impurities from raw water and of agents from water treatment processes^17^. The potential secondary pollution risks of DWTR applied for lake restoration often concerns researchers and lake management organizations. DWTR is commonly considered an inorganic material owing to the high quality of raw water used in a drinking water plant^13^. Particular attention has been directed to the potential risks of metal/metalloid in DWTR. Previous reports have shown that DWTR contain various quantities of arsenic (As), barium (Ba), beryllium (Be), cadmium (Cd), cobalt (Co), chromium (Cr), copper (Cu), manganese (Mn), molybdenum (Mo), nickel (Ni), lead (Pb), and zinc (Zn)^26^,^27^. However, most of them tended to exhibit low concentrations and were largely non-extractable using the European Community Bureau of Reference (BCR) procedure^26^,^27^. The toxicity characteristic leaching procedure (TCLP) recommended by the US Environmental Protection Agency (USEPA) considered DWTR non-hazardous^26^. By contrast, the lability of Co and Mn significantly increased in DWTR after anaerobic incubation^28^. DWTR addition increased the lability of Ba and Mn in soils^29^,^30^ as well as the release potential of As and Cd from river sediments^31^. These contradictory findings suggest the unclear effect of DWTR application on metal/metalloid pollution risks, which hamper the beneficial recycling of DWTR for lake restoration.

Therefore, the metal/metalloid pollution risk of DWTR was comprehensively evaluated in the present study in accordance with the framework presented in Fig. 1. On the one hand, the pollution risks of Al, As, Ba, Be, Cd, Co, Cr, Cu, Fe, Mn, Mo, Ni, Pb, and Zn to lake water and sediments with DWTR addition were investigated under different pH and redox conditions. The quality of lake water was assessed mainly based on human health risk assessment models and relative to regulatory standards; for sediments, the potential risks of the metals and As were analyzed based on fractionation and TCLP assessment methods. On the other hand, bioaccumulation (to *Chironomus plumosus* larvae and *Hydrilla verticillata*) and kinetic luminescent bacteria tests (using *Aliivibrio fischeri*) were applied in combination to evaluate the biological effects of the metals and As in DWTR during application. This study mainly aims to specify the applicability of DWTR for lake internal P loading control and to provide theoretical support for safe recycling of DWTR.

## Results

### Metal/metalloid in lake water

Most of the metals and As concentrations in lake water with and without DWTR addition for 10, 20, and 30 d exhibited minor differences (see Tables S1 and S2 in Supporting Information), indicating that the incubation time used in this study was adequate to investigate the DWTR effect. The mean concentrations of the metals and As in lake water are presented in Fig. 2. Except for undetectable Be, Cd, Co, Cr, and Pb, the other metals and As concentrations in lake water changed to varying degrees under different pH and redox conditions. The effect of DWTR addition on the metals and As concentrations also varied with the changes in conditions (either increased or decreased). A significant increase was observed for Al (*p* < 0.05) under alkaline and aerobic conditions, Fe (*p* < 0.05) and Mn (*p* < 0.01) under acidic conditions, and Mo (*p* < 0.01) under alkaline conditions (n = 3).

### Metal/metalloid extractability in sediments

The results of TCLP analysis for sediments after incubation are shown in Table 1, and the detailed results are presented in Tables S3 and S4 (Supporting Information). The TCLP was designed to determine the mobility of contaminants in liquid, solid, and multiphasic wastes. On the basis of the results of TCLP analysis, hazardousness could be assessed for wastes^32^. Regulatory limits for hazardous waste identification were identified for Ag, As, Ba, Cd, Cr, Hg, Pb, and Se^33^. Among them, only Ba and Cd were detectable in the TCLP leachates of the sediments, whereas the concentrations of Ba (1425–1475 μg L^−1^) and Cd (0.473–0.675 μg L^−1^) were remarkably less than the regulatory limits (Ba: 100000 μg L^−1^ and Cd: 1000 μg L^−1^). These results suggested that the sediments with and without DWTR could be considered non-hazardous. Nonetheless, further comparison indicated that DWTR addition significantly reduced the leachability of Co, Ni (*p* < 0.01), and Mo (*p* < 0.05) but increased the leachability of Mn (*p* < 0.01) (n = 8).

The results of metal and As fractionation in the sediments after incubation are shown in Fig. 3, with the details presented in Figure S1 and Table S4 (Supporting Information). The main fraction of the metals and As in the sediments after DWTR addition did not change. Metal/metalloid found mainly in acid-soluble fraction included Ba (40–49%) and Mn (62–75%). Those in the non-extractable fraction included Al (78–97%), As (88–99%), Be (76–80%), Cd (42–50%), Co (75–77%), Cr (96–98%), Cu (87–93%), Fe (79–90%), Mo (97–100%), Ni (86–87%), Pb (57–82%), and Zn (76–80%). The addition of DWTR significantly increased the acid-soluble fraction of Ba and Mn (*p* < 0.01), as well as the non-extractable fraction of As, Cd, Mo, and Pb (*p* < 0.01). Such addition also decreased the non-extractable fraction of Al, Cu, Fe, and Zn (*p* < 0.01) (n = 8). Sequential extraction procedures used progressively more destructive reagents to provide insight into the association of the solid phases and metal/metalloid^34^. Therefore, DWTR addition significantly increased the lability of Al, Ba, Cu, Fe, Mn, and Zn and decreased the lability of As, Cd, Mo, and Pb in the sediments.

### Biological effect of metal/metalloid

The results of the bioaccumulation test are shown in Fig. 4. For the *C. plumosus* larvae (Fig. 4a), except for As, Be, Cd, Co, and Pb, the other metals could be detected; however, DWTR addition exerted a limited effect on the detectable metals in the larvae (ratio for variation <10% and statistically insignificant). For *H. verticillata* (Fig. 4b), except for Cd and Pb, the other metals and As could be detected; DWTR addition significantly increased Al (*p* < 0.01) and Mn (*p* < 0.01) contents but decreased As (*p* < 0.01), Cu (*p* < 0.05), and Zn (*p* < 0.05) contents in the plant (n = 3). Therefore, DWTR addition changed the biological effect of metals and As; however, the changes were related to specific organisms. In addition, the mortalities of the larvae and the increased weight of plant cultivated in the sediments with and without DWTR were similar, which were, respectively, within 10% and approximately 0.3 g in dry-weight. These results suggest that DWTR addition only slightly affected *C. plumosus* larvae and *H. verticillata* surviving in lake sediments.

The luminescent bacteria toxicity levels of sediments with and without DWTR for acute LI, chronic LI, and growth inhibition are shown in Fig. 5. The sediments without DWTR addition showed acute LI at concentration >50% dilution, and the sediments with DWTR showed acute LI at concentration >12.5% dilution, indicating that DWTR addition enhanced the acute LI of the sediments. Nevertheless, the observed acute LI was <10%; chronic LI and growth inhibition were observed for the sediments with and without DWTR. According to Menz *et al*.^35^, only acute LI >20%, chronic LI >15%, and GI >20% were identified as significant inhibition rates. Therefore, the observed acute toxicity of the sediments with and without DWTR was insignificant and recoverable.

## Discussion

In this study, DWTR addition exhibited dual effects on metal/metalloid concentrations and lability in the water columns (Table 1 and Figs 2 and 3). The reasons may be that the introduction of metals (e.g., Al, Fe, and Mn) with relatively high concentrations or lability from DWTR to sediment (Table S9) caused an increase in lability or release potential (Figs 2 and 3). By contrast, a decrease in the lability of some metals (e.g., Cd, Co, and Pb) in sediments with DWTR was observed because of the immobilization by DWTR^17^. Reasonably, the increases in metal/metalloid lability and concentrations in the environment did not imply that DWTR application for lake restoration would induce harmful effects on the environment. The potential cause of harmful effects has often been identified based on the risk assessment models and the responses of different organisms^36^.

Accordingly, human health risk assessment models were used to determine the potential risk of the metals and As in lake water with DWTR addition (Tables S5, S6, and S7 in Supporting Information). The results indicated that dermal non-carcinogenic risk for the metals and As, as well as oral non-carcinogenic risk for most of the metals and As, in lake water with DWTR were not of concern. DWTR addition even eliminated concerns for As oral non-carcinogenic risk under anaerobic conditions (Table S6) and only slightly affected As oral carcinogenic risks. However, DWTR addition may cause problems for Mn oral non-carcinogenic risk under anaerobic conditions and low pH. A comparison of the metals and As concentrations in lake water with the Environmental Quality Standard for Surface Water in China^37^ and the National Recommended Water Quality Criteria for fresh water in the USA^38^ suggested that the concentrations of Fe under anaerobic conditions, as well as Mn under anaerobic conditions and low pH, remarkably exceeded the standards; the excess was enhanced by DWTR (Fig. 2; further details can be seen in Supporting Information). Therefore, Fe and Mn in lake water exhibited a relatively high pollution risk to the environment under certain conditions, and DWTR addition increased the risks. Notably, the ratio for the amended sediments and lake water was 1:5 in this study. This ratio could be higher than that in the actual environment. This finding suggests that the potential metal/metalloid pollution risks to lake water with DWTR were overestimated in the study.

DWTR addition also caused a significant increase in the leachability of Mn from the sediments (Table 1) and the lability of Al, Ba, Cu, Fe, Mn, and Zn in the sediments (Fig. 3). A statistically significant increase in the mobility of these metals did not indicate a high pollution risk to the environment with DWTR addition. A similar phenomenon is that although Al and Mo concentrations in lake water significantly increased under certain conditions, the concentrations of both metals were far less than those of the regulatory standards (Fig. 2; further details can be seen in the Supporting Information). On the basis of the results of fractionation (Fig. 3), risk assessment codes (RAC) were used to analyze the potential risks of the metals and As in the sediments^39^. The calculated RACs are presented in Table S8 (Supporting Information). DWTR addition did not increase the RAC of the metals in the sediments; instead, it decreased the RAC of As from low risk to no risk under anaerobic conditions. This result was consistent with the TCLP analysis, which demonstrated that the sediments, with and without DWTR, were non-hazardous (Table 1). To verify the finding that DWTR addition only limitedly affected the metals and As pollution risks in the sediments, variations in the biological behaviors of the metals and As in the sediments induced by DWTR addition were further determined based on benthonic animals and aquatic plants (Fig. 4).

Our study suggested that the effect of DWTR addition on the biological behaviors of the metals and As in the sediments varied with specific organisms (Figs 4 and 5). DWTR addition did not induce the enrichment of the metals and As in *C. plumosus* larvae and exerted no effect on the larvae surviving in the sediments (Fig. 4a). Van Alstyne *et al*. also demonstrated that the consumption of land-applied DWTR in a forage system posed no risk to grazing animals (*Ovis aries*) on the basis of a DWTR ingestion test^40^. By contrast, DWTR addition significantly increased Al and Mn contents in *H. verticillata*; however, normal growth of plant was still observed (Fig. 4b). Lombi *et al*. and Mahdy *et al*. found an increased enrichment in Al in *Lactuca sativa* from an acidic and a neutral pH soil and in corn (*Zea mays*) from alkaline soils with DWTR application, respectively^41^,^42^. Meanwhile, Oladeji *et al*. observed no increase in Al content in Bahiagrass or ryegrass *(Lolium perenne* L.) from Immokalee fine sand (Alaquods) applied with DWTR^43^. These differences may be mainly related to the different soil properties and plants used in the tests^42^,^43^. In the study by Novak *et al*., the increase in Mehlich-1–extractable Mn concentrations in soils amended with DWTR was enriched with Mn, the conditions of which potentially caused stress to Mn-sensitive crops^30^. However, similar to our study, no study results have indicated the apparent phytotoxicity of Al and Mn to plants during land application of DWTR^17^. The reasons may be that (i) the release of potentially toxic Al from DWTR was mitigated by its alkaline nature and pH buffering capacity^41^ and that (ii) Al speciation in DWTR would likely be dominated by hydrolysis and organically complexed Al forms rather than free Al^3+^ 17. Limited data were available regarding Mn biological behaviors during DWTR application. The potential effects of Mn and Al on aquatic plants during DWTR application require further investigation.

The limited undesirable effects on aquatic animals and plants obtained in the current study (Fig. 4) was also probably attributable to the tolerance of the selected organisms to the metals and As. Accordingly, based on a relatively more sensitive organism, the luminescent bacteria test was applied to intuitively analyze the possibility of detrimental effects induced by DWTR application. The kinetic luminescent bacteria test applied in this study, which combines the conventional short-term luminescence inhibition test according to EN ISO 11348 and the *Photobacterium phosphoreum* growth inhibition test, allowed the assessment of acute and chronic effects^44^. The *A. fisheri* test conducted in this study has also been considered one of the most common microbial tests used for sediment elutriates^45^. Our study suggests that DWTR addition only caused an insignificant enhancement of sediment acute LI; moreover, the inhibition was recoverable, and neither chronic LI nor growth inhibition were observed for the sediments with and without DWTR (Fig. 5). Therefore, DWTR addition exerted only a slight toxic effect on microorganisms in the sediments. Wang *et al*. reported that DWTR addition could improve the sediment habitats for them to become more appropriate for bacteria survival^20^,^24^,^25^. Ippolito *et al*. also indicated that biosolids and DWTR co-applications did not affect the soil microbial community structure^46^.

In general, DWTR application for lake restoration may neither induce metal/metalloid pollution to sediments nor harm aquatic organisms. However, the finding that DWTR application may cause Mn and Fe concentrations in lake water to exceed regulatory guidelines (although the risks may be overestimated) should be given emphasis. On the basis of the DWTR dosages (Section 2.2), total Fe and Mn contents in DWTR (Table S9), and increased Fe and Mn in overlying water (Fig. 2 and Tables S1 and S2), the maximum release ratio of Mn from DWTR was observed under acidic conditions. The ratio obtained was approximately 3.17% of total Mn in DWTR. Meanwhile, the maximum release ratio of Fe was observed under anaerobic conditions. The ratio obtained was approximately 1.28% of total Fe in DWTR. Therefore, we roughly present a dosage threshold calculation model for safe DWTR application to control eutrophication (Eq. 1) and recommend a procedure for DWTR prescreening (Fig. 6).


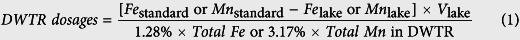


where *Fe*_standard_ or *Mn*_standard_ represents the attainable goal of the water quality guideline; *Fe*_lake_ or *Mn*_lake_, the concentration of Fe or Mn in lake water before eutrophication control; *V*_lake_, the volume of lake water area for restoration; and *Total Fe* or *Total Mn* in DWTR, the total Fe or Mn content in DWTR. In accordance with the model, two calculated dosages are based on Fe and Mn, whereas relatively reduced dosages can be adopted as the dosage threshold for DWTR application. The determined dosage threshold of DWTR was then used for DWTR prescreening (Fig. 6). If the said threshold was higher than the calculated dosages for P immobilization, DWTR was recommended for eutrophication control. The DWTR dosages for P immobilization were mainly calculated based on the mobile P in lake sediment (e.g., NH_4_Cl and Na_2_S_2_O_4_-NaHCO_3_ extractable) and amorphous Al/Fe in DWTR (e.g., oxalate–ammonium oxalate extractable)^47^.

With the exception of Al, Fe, and Mn, other metals and As exerted no effect on the environment. The reason may be that the Al, Fe, and Mn in DWTR applied in this study mainly came from water treatment processes with relatively high concentrations (Table S9), whereas the other metals and As mainly came from the raw water of a water treatment plant and tended to exhibit low concentrations and low lability^27^. The potential detrimental effect of Mn on the environment observed by Novak *et al*. was also for the DWTR produced from a plant, using KMnO_4_ for water purification^30^. From another perspective, land applications of DWTR were found to only slightly affect the accumulation of Cu in *L. sativa*^41^ and despite reports indicating significant decreases in Cd, Ni, Pb, and Cu accumulation in corn (*Z. mays*)^42^. Our study also indicated significant decreases in the leachability of Co, Ni, and Mo (Table 1) from sediments and lability of As, Cd, Mo, and Pb (Fig. 2) in sediments, as well as in the enrichment of As, Cu, and Zn in *H. verticillata* (Fig. 4b). In addition, the effect of DWTR addition on most of the metals and As lability in sediments (Table 1 and Fig. 3) and concentrations in lake water (Fig. 2) was difficult to establish. This result may be attributed to the low concentrations or lability of the metals and As in sediments and DWTR. Therefore, understanding the water treatment processes where DWTR is collected was also important, and emphasis should be given to metal/metalloid in DWTR introduced during water treatment processes in DWTR prescreening.

**Figure 1:**
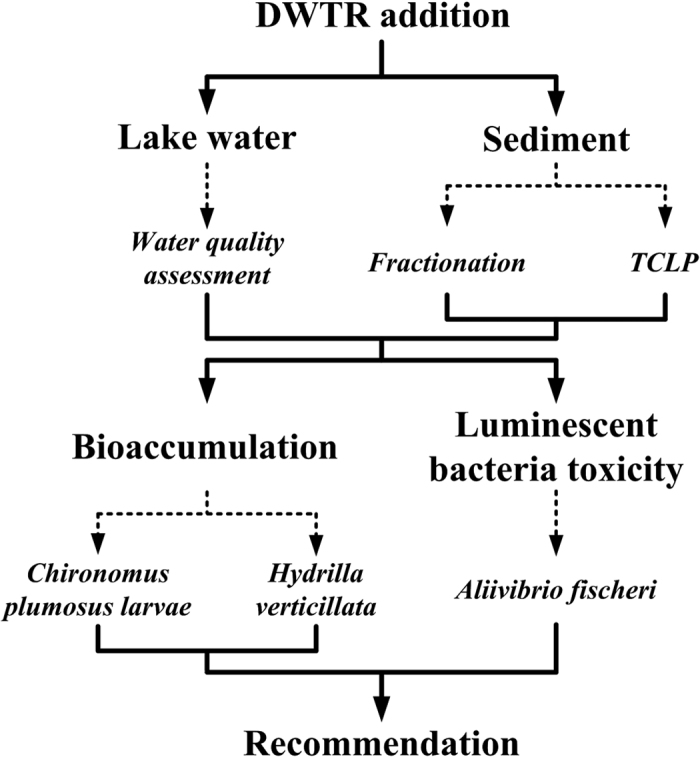
Framework of this study.

**Figure 2:**
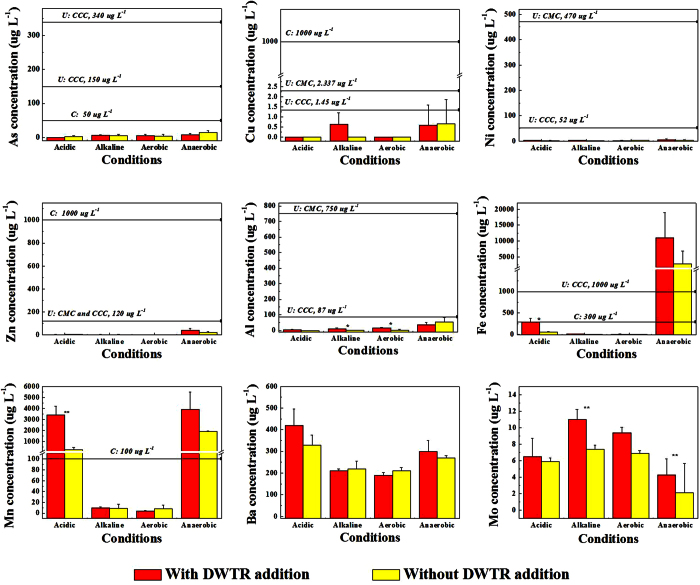
Effect of DWTR addition on the metals and As concentrations in lake water under different conditions. Mean values of concentrations measured at the 10^th^, 20^th^, and 30^th^ d (n = 6) are presented. *And **represent significant differences at *p* < 0.05 and 0.01, respectively, for the metals and As concentrations in lake water with and without DWTR addition. CMC represents Criteria Maximum Concentration; CCC represents Criterion Continuous Concentration; U represents National Recommended Water Quality Criteria for freshwater in USA^37^; C represents Surface Water Quality Standard Class III used in China^38^; and Acute and chronic represent acute and chronic toxicity.

**Figure 3:**
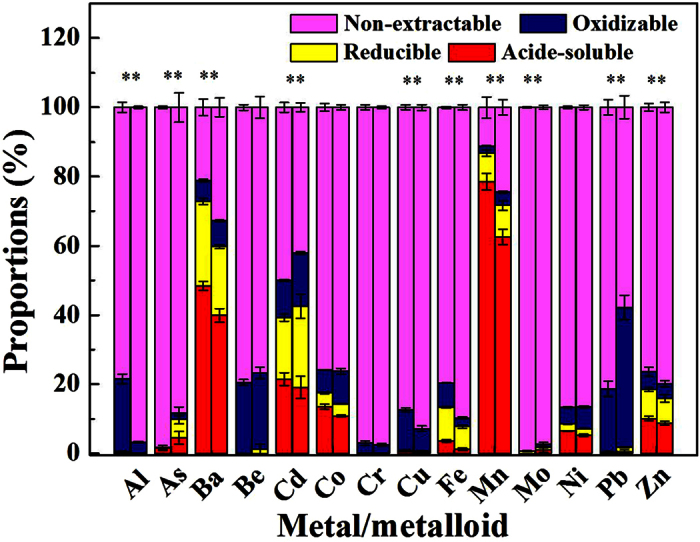
Results of metal and As fractionation in sediments with and without DWTR after incubation. The individual fraction content is the mean content in sediments with or without DWTR after incubation under different conditions. The error bars indicate the variations in each fraction proportion among the 8 samples. For each metal and As, the left column is the element in sediments with DWTR, whereas the right column is the element in sediments without DWTR. *And **represent significant differences at *p* < 0.05 and 0.01, respectively, for metal and As fractions between sediments with and without DWTR.

**Figure 4:**
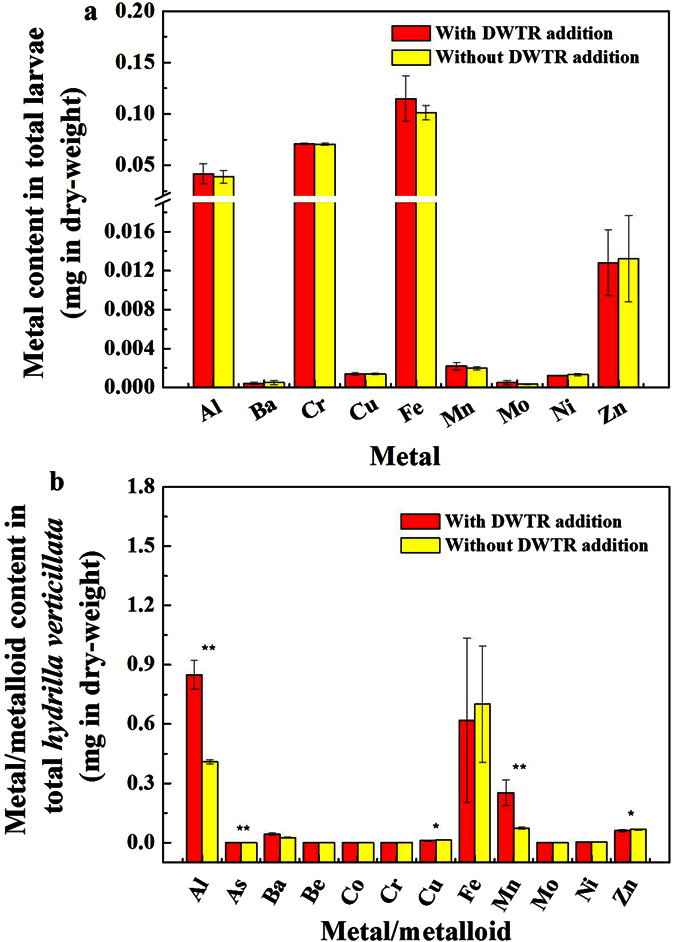
*Chironomus plumosus* larvae bioavailable (**a**) and *Hydrilla verticillata* bioavailable (**b**) metals and As in sediments with and without DWTR (n = 3). *And **represent significant differences at *p* < 0.05 and 0.01, respectively, for the values between sediments with and without DWTR.

**Figure 5:**
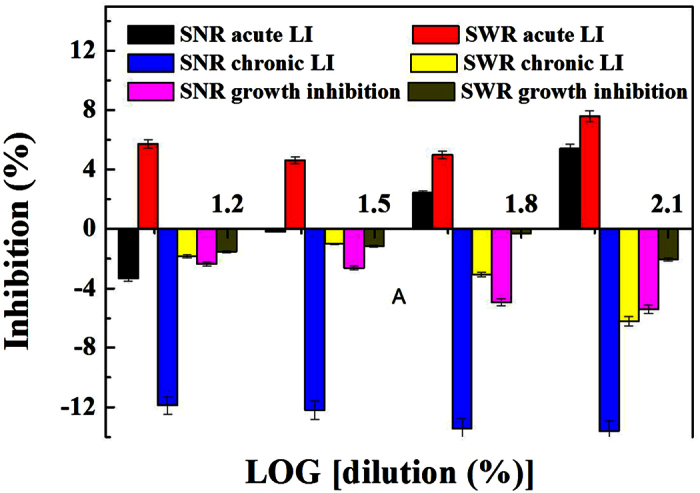
Kinetic luminescent bacteria toxicity of sediments with and without DWTR (n = 3). SNR denotes sediment without DWTR; SWR represents sediment with DWTR.

**Figure 6:**
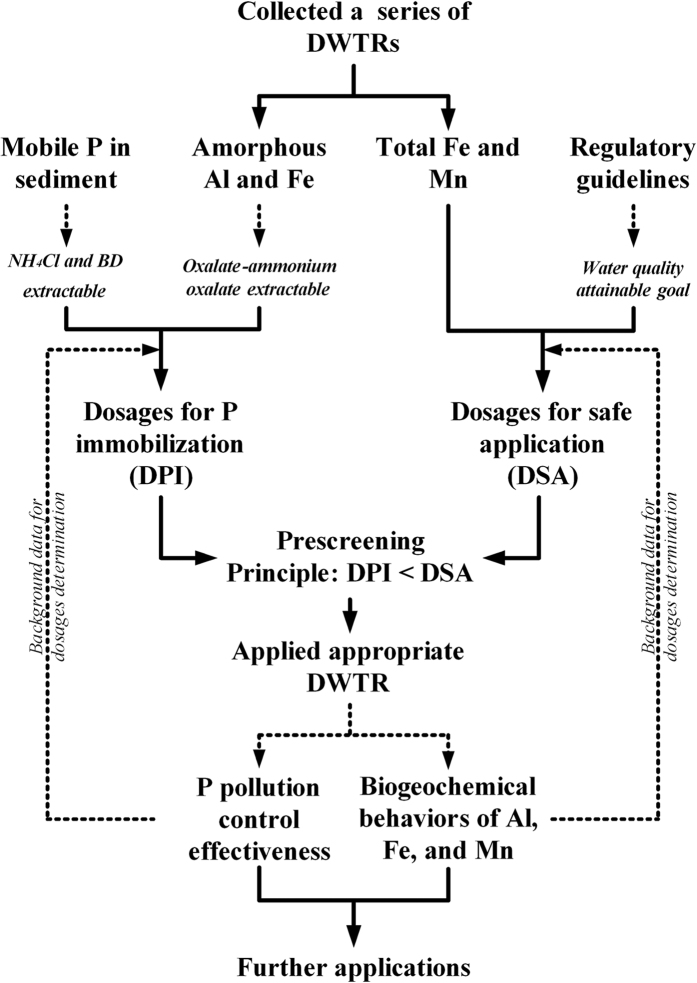
Recommended procedures for DWTR prescreening to control eutrophication. BD denotes Na_2_S_2_O_4_ and NaHCO_3_.

The *in situ* chemical treatment for eutrophication control is generally based on the reactions between metals and P. Accordingly, the reactive materials, which commonly enrich with P inactivating metals (e.g., Al, Ca, Fe, La, or Zr), are often selected for chemical treatment to control eutrophication. Application of the reactive materials may in turn lead to variations in the composition of the lake geochemical background, particularly related to elements with relatively low abundances in Earth’s crust. The regulatory guidelines typically contain information such as the maximum acceptable concentration of contaminants that exhibit no obvious eco-environmental effects, as well as information describing the regional ecosystem and social economical characteristics^36^. Thus, the regulatory guidelines vary to a certain extent for different regions worldwide and different water functions. Consequently, the metal/metalloid pollution risks under various conditions should also be considered as important data to support the use of chemical treatment for eutrophication control apart from control effectiveness. A dosage threshold for the reactive materials should be determined based on the metal/metalloid pollution risks. To the best of our knowledge, the dosage was determined mainly based on the mobile P in lakes and the immobilization capability of the reactive materials. Several reports have demonstrated that metal concentrations in lakes exceeded regulatory standards during lake restoration, although this effect was temporary^8^. Therefore, establishing procedures for metal/metalloid pollution risk assessment and regulating the reactive materials for safe application can benefit the widespread use of chemical treatment for lake restoration. In the current study, the use of different risk assessment methods helped comprehensively understand the metal/metalloid pollution risks for DWTR application (Fig. 1). The results obtained using these methods differed, further indicating that the metal/metalloid pollution risks of reactive materials may vary with different exposure routes. These variations also suggest that pollution risks should be assessed from different perspectives. The pollution risks were evaluated under different pH and redox conditions, given that these two conditions were typical environmental factors affecting the mobility of contaminants in the natural environment^48^. The human health risk assessment models selected in the current study to assess the quality of lake water were used as examples to determine potential risks. In practice, further details could also be associated with the attainable goal of water quality or water functions after restoration (e.g., drinking water sources or scenery-water). *C. plumosus* larvae, *H. verticillata*, and *A. fischeri* were selected, given that these organisms at different trophic levels were most likely to be exposed to DWTR during application (Figs 4 and 5). Finally, we identified a potentially undesirable effect of DWTR application on aquatic ecosystems, presented a dosage threshold calculation model (Eq. 1), and recommended a procedure for DWTR prescreening to ensure safe application (Fig. 6). The scheme employed in this study could also be adopted to assess the safety of other reactive materials. Although composed of inorganic components^13^, DWTR exhibits potential for both adsorbing and releasing organic matter^49^; meanwhile, organic matter is critical for the formation of disinfection byproducts when raw water is used as a drinking water source^50^. Therefore, future studies should also focus on the variation in the properties of organic matter in lake water and sediment with DWTR addition.

## Conclusions

The main conclusions are listed as follows:

1. DWTR addition may pose Mn oral non-carcinogenic risks for lake water under anaerobic conditions and low pH. The concentrations of Fe and Mn under anaerobic conditions as well as low pH markedly exceeded the regulatory standards, and such exceedance was enhanced by DWTR.
2. DWTR addition did not increase the RAC of the metals and As in the sediments; it decreased the RAC of As from low risk to no risk under anaerobic conditions. Moreover, the sediments with and without DWTR can be considered non-hazardous.
3. DWTR addition did not induce the enrichment of the metals and As in *C. plumosus* larvae but significantly increased Al and Mn contents in *H. verticillata*. However, DWTR addition only slightly affected the *C. plumosus* larvae and *H. verticillata* surviving in lake sediments.
4. DWTR addition caused an insignificant increase in sediment acute LI, and the inhibition was recoverable. No chronic LI and growth inhibition for sediments with and without DWTR were observed.

This study finally presented a dosage threshold calculation model and a procedure for DWTR prescreening to ensure safe application. In general, successful DWTR application for eutrophication control should be based on management.

## Materials and Methods

### Sample collection

Samples of sediments were collected from Lake Hengshuihu (37°39′N, 115°39′E) in China. The surface sediments were collected by a grab sampler to a depth of 0–10 cm and then filtered through a 1.8 mm sieve, homogenized, and stored in aseptic valve bags at 4.0 °C. Lake water was collected at the same site at a depth of 0.50 m. The water was filtered using a 0.45 μm Millipore filter paper and then stored at 4.0 °C. Dewatered DWTR was collected from Beijing City No. 9 Waterworks in China. The fresh DWTR was air-dried, ground, and sieved to a diameter less than 1 mm.

The metal/metalloid concentrations in lake water, sediment, and DWTR are presented in Table S9 (Supporting Information). The total contents of silver (Ag), Al, As, Ba, Be, Cd, Co, Cr, Cu, Fe, mercury (Hg), Mn, Mo, Ni, Pb, antimony (Sb), selenium (Se), and Zn in lake sediments and DWTR were measured. However, Ag, Hg, Sb, and Se in the sediments and DWTR could not be detected by inductively coupled plasma–atomic emission spectrometry (ICP–AES, ULTIMA, JY, France). Therefore, except for the 4 undetectable elements, the risks of other metals and As were assessed in this study.

### Incubation test

To evaluate the effect of lake water pH, 100 g of wet sediments were placed into 8 beakers (1 L), with 4 beakers containing 7 g of DWTR and the remaining 4 beakers used as controls. The DWTR represented approximately 10% of the sediments (dry weight)^20^. Approximately 500 mL of lake water was gradually poured into the beakers to avoid solid resuspension. Lake water pH was then maintained within the ranges of 5.5–6.0 and 8.5–9.0, using HCl and NaOH, respectively. Each group included 2 parallel samples, and pH was adjusted daily. The beakers were covered with a gas-permeable film and incubated at 15 °C under dark conditions.

To evaluate the effect of lake water redox conditions, each group included 2 parallel samples, similar to testing for the pH effect. One group was placed into a culture tank. Gas extraction and replacement were then performed 3 times by using the Unijar Suction System (Unitech BioScience Co., Ltd, China) for the tank to create an anaerobic condition. The replacement gas contained N_2_ (80%), CO_2_ (10%), and H_2_ (10%). The other group was covered by a gas-permeable film to maintain an aerobic condition.

In the two tests, lake water was collected every 10 d (the tests lasted 30 d), and the metals and As concentrations were determined by ICP–AES. After incubation, the sediments with and without DWTR were freeze-dried, ground, and sieved to a diameter less than 0.15 mm for further analysis. The properties of lake water during the tests are listed in Table S10 (Supporting Information).

### Solid characterization

BCR sequential extraction was employed to determine the metals and As forms in the sediments after incubation^51^. Sediments were sequentially extracted using 0.11 M CH_3_COOH (pH 2.85), 0.1 M NH_2_OH•HCl (pH 2), and 30% H_2_O_2_ + 1 M CH_3_COONH_4_ (pH 2). This method separates the extracted metals and As into acid-soluble, reducible, and oxidizable fractions. The non-extractable fraction (by the BCR procedure) in the solids were determined as the difference between the sum of each fraction and the total content quantified using USEPA Method 3051^52^. The leachability of the metals and As from the sediments after incubation was measured using the TCLP method^32^. All extracts were filtered using a 0.45 μm micropore filter paper. The metals and As concentrations in the filters were determined by ICP–AES.

### Bioaccumulation test

In this test, *C. plumosus* larva and *H. verticillata* were selected because these organisms are typically found in freshwater aquatic ecosystems. For the *C. plumosus* larva bioaccumulation test, beakers with 50 g of wet sediments were divided into two groups: one group was added with 3.5 g of air-dried DWTR, and the other was used as a control. Deionized water was added accordingly to ensure a water depth of 1–2 cm above the sediments. Subsequently, 40 larvae were added into the beakers. Each beaker was capped with a layer of gauze, and neither aeration nor food was provided^53^. On the 10^th^ d, the larvae were separated from the solids^54^, carefully cleaned with deionized water and allowed to depurate in deionized water for 6 h to empty their digestive tracts^53^. For the *H. verticillata* bioaccumulation test, similar groups were set up for the larva test. Three specimens from *H. verticillata* with approximately 1.5 g fresh weight and 10 cm high were planted in each breaker and then submerged in synthetic fresh water solutions^55^. Harvesting was performed after incubation for 30 d. The harvested plant was carefully cleaned with deionized water and then freeze-dried. The metals and As concentrations in the larvae and plant were determined according to USEPA Method 3051^52^. The organisms were pretreated in accordance with the methods by Xia *et al*.^54^ and Xue *et al*.^55^. Each group had triplicate samples and were incubated at 25 °C with a 16:8 (light:dark) photoperiod^54^.

### Kinetic luminescent bacteria test

The kinetic luminescent bacteria test was conducted based on *A. fischeri (A. fischeri,* previously named *Vibrio fischeri*) in accordance with the method by Menz *et al*.^35^. A pure culture of *A. fischeri* was prepared in supplemented seawater complete media (SSWC media) and incubated overnight (90 rpm, 20 °C). When the turbidity of the bacterial suspension reached 500–700 formazin turbidity units (FTUs), the culture was diluted by SSWC media to an initial turbidity of approximately 20 FTUs. The bacterial suspension, together with SSWC media (blank), was transferred to a 96-well plate. An initial measurement of luminescence and optical density (λ = 578 nm) was performed after pre-tempering for 30 min. The sediment extracts and controls were added. A kinetic measurement of luminescence and optical density was conducted for 24 h by the plate reader (Infinite M200, Tecan, Switzerland) and positioned in a cooling incubator (Thermo Fisher Scientific, USA) at 15 °C. Each sample was tested in triplicate. The raw data were analyzed for three different endpoints, including acute luminescence inhibition (acute LI, 0.5 h), chronic luminescence inhibition (chronic LI, 15 h), and growth inhibition (10 h) relative to the controls.

### Statistical analysis

Data analysis was performed using SPSS version 18.0. For fractionation and TCLP analysis, the relative standard deviation of three parallel sub-samples for each sample was less than 10%. Kolmogorov–Smirnov tests indicated that the data from the replicate samples followed a normal distribution. ANOVA based on α = 0.05 was used to determine the differences in the data obtained.

The detailed descriptions of materials and methods in this study are presented in Supporting Information.

## Additional Information

**How to cite this article**: Yuan, N. *et al*. Applicability of drinking water treatment residue for lake restoration in relation to metal/metalloid risk assessment. *Sci. Rep.* **6**, 38638; doi: 10.1038/srep38638 (2016).

**Publisher's note:** Springer Nature remains neutral with regard to jurisdictional claims in published maps and institutional affiliations.

## Supplementary Material

Supplementary Information

## Figures and Tables

**Table 1:** Results of TCLP analysis for sediments with and without DWTR after incubation and regulatory limits^33^ (μgL^−1^).

Elements	With DWTR	Without DWTR	Regulatory limits
Ba	1425 ± 95.7^a^	1475 ± 50.0	100000
Cd	0.473 ± 0.0727	0.675 ± 0.520	1000
Co**	4.43 ± 0.350	11.8 ± 0.500	—
Cu	16.8 ± 2.36	15.3 ± 1.26	—
Fe	9.75 ± 3.45	6.85 ± 0.834	—
Mn**	**10425** ± **675**	**6875** ± **171**	—
Mo*	8.70 ± 2.13	22.8 ± 8.26	—
Ni**	13.3 ± 2.63	18.8 ± 0.500	—
Zn	27.8 ± 17.6	45.8 ± 15.8	—

^a^Represents mean ± standard error, n = 8; ^*^and ^**^represent significant differences at P < 0.05 and 0.01, respectively, for the data obtained with and without DWTR.
