# Supplementary material for: Applicability of drinking water treatment residue for lake restoration in relation to metal/metalloid risk assessment
Source: Sci Rep. 2016 Dec 8;6:38638. doi: 10.1038/srep38638 (PMC5144140; doi:10.1038/srep38638)
Supplement: Supplementary Information [file srep38638-s1.pdf]

Supporting Information for

Applicability of drinking water treatment residue for lake restoration in relation to metal/metalloid risk assessment

Nannan Yuan[1,2], Changhui Wang[1,2,*], Yuansheng Pei[2,*], Helong Jiang[1]

[1] *State Key Laboratory of Lake Science and Environment, Nanjing Institute of Geography and Limnology, Chinese Academy of Sciences, Nanjing 210008, China*

[2] *The Key Laboratory of Water and Sediment Sciences, Ministry of Education, School of Environment, Beijing Normal University, Beijing 100875, P. R. China*

[*] Corresponding Author: Tel/fax: 86-10-5880 1830; E-mail address: yspei@bnu.edu.cn (Y.S. Pei); chwang@niglas.ac.cn (C.H. Wang)

Materials and methods

Sample collection

Lake Hengshuihu is the second largest fresh water lake in north China plain, covering an area of 283 km^2 and also is the only nature reserve that contains swamp, waters, beach, meadow, and forest in north China plain. The average depth of the lake is approximately 4–5 m, the annual average temperature is 13 ℃, and the annual rainfall is 518.9 mm. The Yellow river is irregularly used as water source to supply the lake during dry season. Sediments were sampled from Lake Hengshuihu (37°39′N, 115°39′E) in September 2012. The surface sediments were collected by a grab sampler to a depth of 0–10 cm, and then were filtered through a 1.8-mm sieve, homogenized, and stored in aseptic valve bags at 4.0 ℃. Lake water was collected at the same site at a depth of 0.50 m. The water was filtered using a 0.45-μm Millipore filter paper and then stored at 4.0 ℃.

Dewatered DWTR was collected from Beijing City No. 9 Waterworks in China in September 2012. In this facility, both surface water and groundwater are used as drinking water sources. Coagulation-precipitation-filtration is used for conventional water treatment, and activated carbon adsorption is used for advanced water treatment. Moreover, polyaluminum chloride and polyferric chloride are used as coagulants. The fresh DWTR was air-dried, ground, and sieved to a diameter less than 1 mm. The metal/metalloid concentrations of the lake water, sediment, and DWTR are presented in Table S9.

The total contents of Ag, Al, As, Ba, Be, Cd, Co, Cr, Cu, Fe, Hg, Mn, Mo, Ni, Pb, Sb, Se, and Zn in lake sediments and DWTR were measured. However, the total contents of Ag, Hg, Sb, and Se in the sediments and DWTR were below the detection limits of inductively

coupled plasma-atomic emission spectrometry (ICP-AES, ULTIMA, JY, France). Therefore, except the four undetectable elements, the risks of other metals and As were assessed herein.

Incubation test

To examine the effect of lake water pH, 100 g of wet sediments was placed into eight beakers (1 L). Four beakers contained 7 g of air-dried DWTR, while the other four beakers were used as controls. The DWTR represented approximately 10% of the sediments in dry weight[1]. Lake water of 500 mL was added to the beakers slowly to avoid solids resuspension. Next, lake water pH was kept at 5.5–6.0 and 8.5–9.0, respectively, using either HCl or NaOH. Each group has two parallel samples and pH was daily adjusted. The beakers were covered with a gas-permeable film and incubated at 15 ℃ in dark.

To examine the effect of lake water redox conditions, similar to the test of the pH effect, each group had two parallel samples. One group was put into a culture tank. Then, the gas extraction and replacement processes were performed three times by a Unijar Suction System (Unitech BioScience Co., Ltd, China) for the tank to create anaerobic condition. The replacement gas contained N_2 (80%), CO_2 (10%), and H_2 (10%). The other was covered by a gas-permeable film to maintain aerobic condition.

In the above two tests, 10 mL lake water was collected every 10 d (the tests lasted 30 d), and the metals and As concentrations were determined using ICP-AES. After incubation, the sediments with and without DWTR were freeze-dried, ground, and sieved to a diameter less than 0.15 mm for further analysis. The properties of lake water during the tests can be seen in Table S10.

Solid characterization

The European Community Bureau of Reference (BCR) sequential extraction procedure was used to determine the metals and As forms in the sediments with and without DWTR[2]. Sediments were sequentially extracted using 0.11 M CH_3COOH (pH 2.85), 0.1 M NH_2OH•HCl (pH 2), and 30% H_2O_2 + 1 M CH_3COONH_4 (pH 2). The BCR method separates the extracted metals into acid-soluble, reducible, and oxidizable fractions. The non-extractable fraction (by the BCR procedure) were calculated as the difference between the sum of each fraction and the total content quantified using USEPA Method 3051[3]. The leachability of the metals and As from the sediments was measured using the TCLP method[4]. All extracts were filtered using 0.45-μm micropore filter paper, and the metals and As concentrations in the filtered extracts were determined using ICP-AES.

Bioaccumulation test

In this test, *chironomus plumosus* larva and *hydrilla verticillata* were selected because they were typical organisms in freshwater aquatic ecosystems. For *chironomus plumosus* larva bioaccumulation test, 6 beakers with 50 g wet sediments were evenly divided into two groups. One group was added with 3.5 g air-dried DWTR, and the other was as control. Deionized water was added accordingly to ensure a water depth of 1 to 2 cm above the sediments. Next, 40 larvae were added to the beakers. Each beaker was capped with a layer of gauze, and neither aeration nor food was provided[5]. On the 10th d, the larvae were separated from the sediments[6]. The larvae were removed from the sediment by sieving, carefully

cleaned with deionized water, and allowed to depurate in deionized water for 6 h to empty their digestive tracts[5]. For *hydrilla verticillata* bioaccumulation test, similar groups were set as the larva's test. Three *hydrilla verticillata* with approximately 1.5 g fresh weight and 10 cm high were planted in each breaker and submerged in synthetic fresh water solutions[7]. Harvesting was performed after 30 d incubation. The harvested plant was carefully cleaned with deionized water and oven-dried. The metals and As concentrations in the larvae and plant were determined according to USEPA Method 3051[3]. In addition, the larvae and plant were collected from Lake Hengshuihu. The pretreatments of the larvae and plant was done according to Xia *et al.*[6] and Xue *et al.*[7]. Briefly, the larvae were acclimated to laboratory conditions in deionized water for 2 d to purge their guts, and the larvae in uniform size were selected for the tests. After collecting the *hydrilla verticillata*, young shoots were separated from the mother plant and plants were acclimatized for 7 d in synthetic fresh water solutions prior to the experiment. The composition of the synthetic fresh water was (mg L^{-1}): 22.7 $MgSO_4 \cdot 7H_20$, 30.7 $MgCl_2 \cdot 2H_2O$, 20.4 $CaCl_2 \cdot 2H_2O$, 45.7 NaCl, 26.0 $NaHCO_3$, 3.61 KCl, 1.41 $FeCl_3 \cdot 6H_2O$, 0.97 $Al_2(SO_4)_3 \cdot 18H_2O$, 0.19 $MnCl_2 \cdot 4H_2O$, and (μg L^{-1}): 3.86 $ZnSO_4 \cdot 7H_2O$, 2.17 $CuCl_2 \cdot 2H_2O$ (pH adjusted to 7.0 with NaOH or HCl solutions). All groups were incubated at 25 ℃ with a 16:8 (light:dark) photoperiod[6].

The kinetic luminescent bacteria test

The kinetic luminescent bacteria was carried out based on the marine bacteria, *Aliivibrio fischeri* (*A. fischeri,* named *Vibrio fischeri* before) according to Menz *et al.*[8]. The freeze-dried luminescent bacteria were purchased from Hamamatsu Photonics (Beijing, China) and stored

till usage at -20 ℃. Briefly, a pure culture of *A. fischeri* was prepared in supplemented seawater complete media (SSWC media) and incubated over night (90 rpm, 20 ℃). After the turbidity of the bacteria suspension reached 500–700 formazin turbidity units (FTU), the culture was diluted with SSWC media to an initial turbidity of 20 FTU approximately. The bacteria suspension and SSWC media (blank) was transferred to a 96-well plate, and an initial measurement of luminescence and optical density (λ=578 nm) was performed after pre-tempered for 30 min. Subsequently, the sediment extracts and controls were added, and a kinetic measurement of luminescence and optical density was conducted for 24 h by the plate reader (infinite M200, Tecan, Switzerland) and positioned in a cooling incubator (Thermo Fisher Scientific, USA) at 15 ℃. Each sample was tested in triplicate.

Test solution preparation: Aqueous extracts of samples were prepared according to Ocampo-Duque *et al.*[9] with some modification. Sample of 3 g was mixed with 30 mL of 3% (w/v) aq. NaCl solution, shaking for 12 h at 20 ℃, and then filtering on 0.45 um pore diameter membrance filters.

Sediment preparation: The DWTR were mixed with sediment at dosages accounting for 0 (control) and 10% of sediment in dry weight. The mixtures were incubated for 10 d[1]. After incubation, the mixtures were freeze-dried, ground, and sieved to a diameter less than 1 mm.

In the untreated cultures, the transition between the exponential and the stationary growth phase was reached after approximately 10 h, and the maximum luminescence was reached in the late stationary growth phase after approximately 16 h. Accordingly, 10 and 15 h could be the suitable exposure time for the analysis of the growth inhibition and chronic luminescence inhibition compared to the controls, respectively. The acute luminescence

inhibition was measured after the bacteria were exposed 30 min.

Statistical analysis

Data analysis was performed using SPSS version 18.0. For fractionation and TCLP analysis, the relative standard deviation of three parallel sub-samples for each sample was less than 10%. Kolmogorov-Smirnov tests indicated that the data from the replicate samples followed a normal distribution. Therefore, one-way analysis of variance (ANOVA), based on $\alpha = 0.05$, was used to determine the differences of data obtained accordingly.

Table S1 The detailed results of the metals and As concentrations in lake water under different pH (ug L^{-1}).

pH	5.5-6.0					
Time (d)	10		20		30	
Sediments	With DWTR	Without DWTR	With DWTR	Without DWTR	With DWTR	Without DWTR
Al	<DL[a]	<DL	2.5 ± 2.6	<DL	6.7 ± 1.8	<DL
As	<DL	2.0 ± 2.0	<DL	<DL	<DL	5.1 ± 5.1
Ba	350 ± 10[b]	280 ± 8.6	420 ± 19	330 ± 0.74	500 ± 9.8	370 ± 0.82
Be	<DL	<DL	<DL	<DL	<DL	<DL
Cd	<DL	<DL	<DL	<DL	<DL	<DL
Co	<DL	<DL	<DL	<DL	<DL	<DL
Cr	<DL	<DL	<DL	<DL	<DL	<DL
Cu	<DL	<DL	<DL	<DL	<DL	<DL
Fe	380 ± 39	55 ± 8.0	300 ± 7.0	65 ± 8.4	200 ± 0.31	46 ± 2.3
Mn	2700 ± 59	150 ± 2.5	3200 ± 190	170 ± 63	4300 ± 11	480 ± 40
Mo	9.0 ± 1.2	5.6 ± 0.79	5.1 ± 0.49	6.4 ± 0.29	5.3 ± 0.17	5.8 ± 0.20
Ni	3.0 ± 0.17	2.7 ± 0.74	1.9 ± 0	<DL	2.8 ± 0.25	1.6 ± 1.0
Pb	<DL	<DL	<DL	<DL	<DL	<DL
Zn	2.0 ± 0.16	2.9 ± 0.39	2.4 ± 0.80	2.6 ± 0.63	5.5 ± 1.2	5.4 ± 0.35

P	32±2.0	44±6.7	30±1.6	45±1.1	29±2.4	48±14
pH	8.5-9.0					
Time (d)	10		20		30	
Sediments	With DWTR	Without DWTR	With DWTR	Without DWTR	With DWTR	Without DWTR
Al	16±3.4	<DL	8.9±2.4	2.5±2.5	7.8±1.2	<DL
As	7.9±0.35	7.9±0.48	4.9±4.9	7.0±2.1	<DL	<DL
Ba	200±7.5	180±2.1	210±4.0	220±11	220±5.0	250±6.2
Be	<DL	<DL	<DL	<DL	<DL	<DL
Cd	<DL	<DL	<DL	<DL	<DL	<DL
Co	<DL	<DL	<DL	<DL	<DL	<DL
Cr	<DL	<DL	<DL	<DL	<DL	<DL
Cu	<DL	<DL	1.1±1.1	<DL	0.81±0.81	<DL
Fe	24±9.5	2.0±0.27	8.6±1.8	5.3±1.4	6.0±1.6	8.8±4.8
Mn	12±3.2	1.4±0.68	8.8±6.0	7.5±0.87	10±4.5	17±14
Mo	12±0.70	8.0±0.32	12±0.76	7.2±0.18	9.9±0.01	7.1±0.36
Ni	2.8±0.23	2.1±0.060	3.5±1.5	2.2±0.14	2.4±0.16	2.0±0.33
Pb	<DL	<DL	<DL	<DL	<DL	<DL
Zn	0.83±0.33	0.044±0.044	4.4±2.4	1.8±0.72	1.6±0.54	3.8±2.4
P	37±4.8	47±6.4	35±0.58	52±7.5	31±2.4	38±4.6

[a] Below the detection limit;

[b] Mean ±standard deviation, n=2.

Table S2 The detailed results of the metals and As concentrations in lake water under aerobic and anaerobic conditions (ug L^{-1}).

Condition	Aerobic					
Time (d)	10		20		30	
Sediments	With DWTR	Without DWTR	With DWTR	Without DWTR	With DWTR	Without DWTR
Al	19 ±5.5[a]	<DL[b]	13 ±2.4	<DL	15 ±1.6	7.8 ±1.4
As	9.3 ±0.74	<DL	3.1 ±3.1	10 ±1.1	3.2 ±3.2	<DL
Ba	200 ±2.3	200 ±2.3	180 ±1.2	210 ±2.2	200 ±3.3	230 ±6.1
Be	<DL	<DL	<DL	<DL	<DL	<DL
Cd	<DL	<DL	<DL	<DL	<DL	<DL
Co	<DL	<DL	<DL	<DL	<DL	<DL
Cr	<DL	<DL	<DL	<DL	<DL	<DL
Cu	<DL	<DL	<DL	<DL	<DL	<DL
Fe	17 ±11	1.2 ±0.10	3.1 ±1.6	1.9 ±0.31	3.9 ±1.7	5.5 ±0.76
Mn	3.5 ±1.1	0.62 ±0.16	2.6 ±1.0	12 ±7.1	4.9 ±3.6	12 ±6.2
Mo	8.7 ±0.27	6.8 ±0.46	10 ±0.62	7.3 ±0.020	9.6 ±0.26	6.7 ±0.075
Ni	2.6 ±0.60	4.0 ±1.0	2.7 ±0.10	1.8 ±0.085	1.7 ±0.080	2.0 ±0.19
Pb	<DL	<DL	<DL	<DL	<DL	<DL
Zn	0.50 ±0.50	0.83 ±0.15	<DL	0.47 ±0.47	<DL	0.25 ±0.25
P	37 ±2.8	62 ±23	34 ±4.6	54 ±2.8	30 ±1.8	42 ±14
Condition	Anaerobic					

Time (d)	10		20		30	
Sediments	With DWTR	Without DWTR	With DWTR	Without DWTR	With DWTR	Without DWTR
Al	54±1.4	87±22	28±8.5	28±11	26±4.7	47±3.7
As	9.0±2.1	18±11	11±3.1	18±43	2.9±2.9	8.8±1.9
Ba	250±4.2	280±2.3	300±17	270±2.2	350±17	260±23
Be	<DL	<DL	<DL	<DL	<DL	<DL
Cd	<DL	<DL	<DL	<DL	<DL	<DL
Co	<DL	<DL	<DL	<DL	<DL	<DL
Cr	<DL	<DL	<DL	<DL	<DL	<DL
Cu	<DL	<DL	<DL	<DL	0.0018± 0.00017	0.0020± 0.00013
Fe	4700±370	7500±59	8800±4800	690±65	20000±4300	280±55
Mn	2300±130	2.0±3.7	3800±480	1900±100	5500±380	1900±480
Mo	5.8±0.040	<DL	4.9±0.66	6.2±6.2	2.2±0.23	<DL
Ni	8.4±2.1	5.9±1.6	4.1±0.88	3.4±0.87	1.8±0.12	1.9±0.15
Pb	<DL	<DL	<DL	<DL	<DL	<DL
Zn	51±26	28±18	20±7.7	12±4.3	48±17	22±37
P	56±0.54	790±13	59±0.49	1200±1.6	78±1.5	1200±2.6

[a] Mean ± standard deviation, n=2;

[b] Below the detection limits.

The TCLP analysis results

Table S3 The results of TCLP analysis for sediments with and without DWTR after incubation test and the regulatory limits[10] (ug L^{-1}).

Conditions	pH				Regulatory limits
	5.5–6.0		8.5–9.0		
Elements	With DWTR	Without DWTR	With DWTR	Without DWTR	
Ba	1300±58[a]	1500±9.2	1500±57	1400±12	100000
Cd	0.44±0.01	7.5±0.02	0.58±0.06	0.73±0.08	1000
Co	3.9±0.24	11±0.08	4.6±0.76	12±0.37	-
Cu	15±0.73	14±1.4	44±34	15±1.1	-
Fe	7.7±52	6.4±0.90	14±2.5	8.1±1.8	-
Mn	10000±700	7100±130	11000±720	6800±490	-
Mo	6.6±0.03	19±1.8	10±0.73	35±22	-
Ni	11±1.1	18±0.13	17±7.0	19±0.46	-
Zn	10±2.0	35±3.9	25±12	42±0.008.7	-

Conditions	Redox conditions				Regulatory limits
	Aerobic		Anaerobic		
Elements	With DWTR	Without DWTR	With DWTR	Without DWTR	
Ba	1400±23	1500±20	1500±35	1500±1.2	100000
Cd	0.42±0.01	0.66±0.09	0.45±0.04	0.61±0.01	1000

Co	4.6±0.21	12±0.12	4.6±0.16	12±0.21	-
Cu	15±2.4	15±0.06	17±0.04	17±8.5	-
Fe	6.3±1.2	6.4±0.28	11±3.9	6.5±0.09	-
Mn	11000±900	6900±78	9700±110	6700±98	-
Mo	7.2±0.41	20±0.28	11±2.3	17±2.3	-
Ni	12±0.33	19±1.2	13±0.06	19±0.12	-
Zn	24±6.4	37±2.2	52±3.6	69±1.2	-

[a] Mean ± standard deviation, n=2.

The extractability of the metals and As in raw sediments.

Table S4 The extractability of the metals and As in raw sediments.

Elements	Fractionation (%)				TCLP
	Acid-soluble	Reducible	Oxidizable	Non-extractable	(ug L^{-1})
Al	0.028	0.12	3.1	97	<DL[a]
As	4.3	6.0	4.1	86	<DL
Ba	40	21	7.7	31	1500
Be	<DL	3.1	22	75	<DL
Cd	14	24	13	48	0.57
Co	9.9	3.7	9.8	77	13
Cr	0.025	0.11	3.7	96	<DL
Cu	0.32	0.36	7.6	92	16
Fe	0.48	7.0	2.7	90	15
Mn	62	11	4.1	22	6800
Mo	1.0	<DL	1.2	98	18
Ni	4.9	2.1	6.4	87	21
Pb	<DL	1.1	40	59	<DL
Zn	7.3	7.7	6.3	79	38

[a] Below the detection limit.

Human health risk assessment for metal/metalloid in lake water

A human health risk assessment model was used to determine the potential the metal and As risk of DWTR addition to lake water. The assessments can be divided into non-carcinogenic and carcinogenic risks, which are based on the oral and dermal exposure routes. The detailed calculation methods are presented below. Normally, the potential non-carcinogenic risk is of concern when the hazard quotient (*HQ*) or hazard index (*HI*) exceeds 1[11]. Carcinogenic risk is the probability of an individual developing any type of cancer from the lifetime exposure to a carcinogen. The acceptable or tolerable risk for regulatory purposes is in the range of 10^{-6} to 10^{-4}[11].

For the non-carcinogenic effects, it can be calculated using the following equations[12,13].

$$HQ_{\mathrm{oral}} = \frac{D_{\mathrm{oral}}}{RfD_{\mathrm{o}}} \quad (1)$$

$$HQ_{\mathrm{dermal}} = \frac{D_{\mathrm{dermal}}}{RfD_{\mathrm{dermal}}} \quad (2)$$

$$HI = \sum HQ \quad (3)$$

$$RfD_{\mathrm{dermal}} = RfD_{\mathrm{o}} \times ABS_{\mathrm{GI}} \quad (4)$$

$$D_{\mathrm{oral}} = \frac{C_{\mathrm{w}} \times IR \times ED \times EF}{BW \times AT} \quad (5)$$

$$D_{\mathrm{dermal}} = \frac{K_{\mathrm{p}} \times C_{\mathrm{w}} \times t_{\mathrm{event}} \times EV \times ED \times EF \times SA}{BW \times AT} \quad (6)$$

where, *AT*, average time (days) (carcinogenic effects AT = 70 years$\times$365 days years^{-1} and non-carcinogenic effects $AT = ED\times$365 days years^{-1}); *BW*, body weight (70 kg); C_{w}, chemical

concentration in water (mg cm$^{3\ -1}$) - chemical-specific values; D_{oral}, oral dose (mg kg^{-1} day^{-1}); D_{dermal}, dermal absorbed dose (mg kg^{-1} day^{-1}); *ED*, exposure duration (70 years); *EF*, exposure frequency (350 days year^{-1}); *EV*, event frequency (1 events day^{-1}); *HI*, hazard index for multiple substances and/or exposure pathways; HQ_{dermal}, dermal hazard quotient; HQ_{oral}, oral hazard quotient; *IR*, oral rate per unit time (2 L day^{-1}); K_p, dermal permeabllity coefficient of compound in water (cm h^{-1}) - chemical-specific values; RfD_{dermal}, reference dose dermal (mg kg^{-1} day^{-1}); RfD_o, reference dose oral (mg kg^{-1} day^{-1}); *SA*, skin surface area available for contact (18000 cm^2); t_{event}, event duration (0.58 h event^{-1}). The *AT*, *BW*, *EF*, *EV*, *SA* and t_{event} are referred to USEPA[12,13]; the *ED* is referred to Hamidin *et al.*[14]; the RfD_o is referred to USEPA[15]. The calculated results can be seen in Tables S7 and S8.

For the cancer risk， it can be calculated using the following equations[12,13].

$$Oral\ cancer\ risk = D_{oral} \times SF_{ABS} \quad (7)$$

$$Dermal\ cancer\ risk = D_{dermal} \times SF_{ABS} \quad (8)$$

$$SF_{ABS} = \frac{SF_o}{ABS_{GI}} \quad (9)$$

where ABS_{GI}, gastrointestinal absorption; SF_{ABS}, absorbed slope factor; SF_o, oral slope factor (mg kg^{-1} day^{-1}). The ABS_{GI} are referred to USEPA;[3,4] the SF_o is referred to USEPA[15]. The calculated results can be seen Table S9.

The *HQ* for the metal and As in dermal exposure (HQ_{dermal}) under different pH and redox conditions was below 1 (Table S8), indicating that dermal non-carcinogenic risk for each metal and As in lake water with DWTR addition was not of concern. For the *HQ* in oral exposure (HQ_{oral}), with the exceptions of As and Mn, the values for the other metals were also below 1. In comparison, HQ_{oral} exceeded 1 for Mn in the overlying lake water with DWTR

addition under anaerobic condition and pH 5.5–6.0, and that for Mn and As in lake water without DWTR addition under anaerobic condition. These results indicated that DWTR addition did not cause concerns for the oral non-carcinogenic risk for most metals in lake water and can even eliminate the concerns for As risk under anaerobic condition. However, at low pH, the addition may cause concerns for Mn oral non-carcinogenic risk.

The *HI* for the metal and As by dermal and oral exposure (HI_{dermal} and HI_{oral}) is the sum of HQ_{dermal} and HQ_{oral} for each metal and As in lake water (Table S8). The HI_{dermal} values for the metal and As in lake water under different conditions were below 1, indicating that dermal non-carcinogenic risk for the metal and As in lake water with DWTR addition was not of concern. However, the HI_{oral} for the metal and As exceeded 1 in the overlying lake water with DWTR addition under anaerobic condition and pH 5.5–6.0, and without DWTR addition under anaerobic condition, while for other conditions, the HI_{oral} was below 1. These results demonstrated that the oral non-carcinogenic risk for the metal and As in lake water with and without DWTR addition may be of concern. Further calculation showed that the HQ_{oral} for Mn accounted for 95 and 79% of the HI_{oral} for lake water with DWTR addition under anaerobic condition and pH 5.5–6.0, while for lake water without DWTR addition under anaerobic condition, the HQ_{oral} for As and Mn accounted for 37 and 58% of the HI_{oral}, respectively. Therefore, DWTR addition, on the one hand, increased HI_{oral} by increasing Mn HQ_{oral}, heightening the concerns for oral non-carcinogenic risk of metal/metalloid in lake water under anaerobic condition and low pH. On the other hand, the addition could reduce HI_{oral} by decreasing As HQ_{oral}, alleviating the concerns for oral non-carcinogenic risk under anaerobic condition.

In this study, among detectable metal/metalloid in lake water, only As had a carcinogenic risk (Table S9). The As dermal carcinogenic risks in lake water with and without DWTR addition were in the acceptable range (< 3.4×10^{-6}). The As oral carcinogenic risks in lake water were mostly not acceptable, and the risks for As with and without DWTR addition were in the same order of magnitude (1.4×10^{-4} to 6.2×10^{-4}). The only exception was in the case of a pH range of 5.5 to 6.0, in which the As oral risk was < 9.9×10^{-5}. These results suggested that DWTR addition could not reduce the As oral carcinogenic risks to an acceptable range for lake water.

Table S5 The calculated oral and dermal dose in this study.

Conditions	pH 5.5–6.0				pH 8.5–9.0			
	With DWTR		Without DWTR		With DWTR		Without DWTR	
Element	$D_{ingstion}$	D_{dermal}	D_{oral}	D_{dermal}	$D_{ingstion}$	D_{dermal}	D_{oral}	D_{dermal}
Al	8.5E-05	4.4E-07	NC	NC	3.0E-04	1.6E-06	2.3E-05	1.2E-07
As	NC[a]	NC	6.6E-05	3.4E-07	1.7E-04	8.9E-07	1.4E-04	7.2E-07
Ba	1.2E-02	6.0E-05	9.0E-03	4.7E-05	5.8E-03	3.0E-05	6.0E-03	3.1E-05
Cu	NC[a]	NC	NC	NC	1.8E-05	9.2E-08	NC	NC
Fe	7.9E-03	4.1E-05	1.5E-03	7.9E-06	3.6E-04	1.9E-06	1.5E-04	7.7E-07
Mn	9.3E-02	4.9E-04	7.4E-03	3.9E-05	2.7E-04	1.4E-06	2.4E-04	1.2E-06
Mo	1.8E-04	9.3E-07	1.6E-04	8.4E-07	3.0E-04	1.6E-06	2.0E-04	1.1E-06
Ni	7.1E-05	7.4E-08	3.8E-05	4.0E-08	7.9E-05	8.3E-08	5.8E-05	6.0E-08
Sr	4.7E-02	2.4E-04	5.2E-02	2.7E-04	2.7E-02	1.4E-04	3.6E-02	1.9E-04
V	NC	NC	NC	NC	7.9E-06	4.1E-08	2.1E-05	1.1E-07
Zn	8.2E-05	2.6E-07	9.9E-05	3.1E-07	6.3E-05	2.0E-07	5.2E-05	1.6E-07
Conditions	Aerobic condition				Anaerobic condition			
	With DWTR		Without DWTR		With DWTR		Without DWTR	
Element	$D_{ingstion}$	D_{dermal}	D_{oral}	D_{dermal}	$D_{ingstion}$	D_{dermal}	D_{oral}	D_{dermal}
Al	4.4E-04	2.3E-06	7.1E-05	3.7E-07	9.9E-04	5.1E-06	1.5E-03	7.7E-06
As	1.4E-04	7.4E-07	9.0E-05	4.7E-07	2.1E-04	1.1E-06	4.1E-04	2.1E-06
Ba	5.2E-03	2.7E-05	5.8E-03	3.0E-05	8.2E-03	4.3E-05	7.4E-03	3.9E-05

Cu	NC	NC	NC	NC	1.6E-05	8.6E-08	1.8E-05	9.6E-08
Fe	2.2E-04	1.1E-06	7.9E-05	4.1E-07	3.0E-01	1.6E-03	7.7E-02	4.0E-04
Mn	1.0E-04	5.3E-07	2.2E-04	1.2E-06	1.1E-01	5.6E-04	5.2E-02	2.7E-04
Mo	2.6E-04	1.3E-06	1.9E-04	9.9E-07	1.2E-04	6.1E-07	5.8E-05	3.0E-07
Ni	6.3E-05	6.6E-08	7.1E-05	7.4E-08	1.3E-04	1.4E-07	1.0E-04	1.1E-07
Zn	4.7E-06	1.5E-08	1.4E-05	4.5E-08	1.1E-03	3.4E-06	5.8E-04	1.8E-06

[a] Data cannot be calculated because the metal concentration in lake water was below detection limit.

Table S6 Non-carcinogenic risks (HQ) for the metals and As in overlying lake water of sediments with and without DWTR.

Conditions	pH 5.5-6.0				pH 8.5-9.0			
	With DWTR		Without DWTR		With DWTR		Without DWTR	
Element	HQ_{oral}[a]	HQ_{dermal}[b]	HQ_{oral}	HQ_{dermal}	HQ_{oral}	HQ_{dermal}	HQ_{oral}	HQ_{dermal}
Al	8.5E-05	4.4E-07	NC	NC	3.0E-04	1.6E-06	2.3E-05	1.2E-07
As	NC[c]	NC	2.2E-01	1.2E-03	5.7E-01	3.1E-03	4.6E-01	2.5E-03
Ba	5.8E-02	4.3E-03	4.5E-02	3.4E-03	2.9E-02	2.1E-03	3.0E-02	2.2E-03
Cu	NC	NC	NC	NC	4.4E-04	2.3E-06	NC	NC
Fe	1.1E-02	5.9E-05	2.2E-03	1.1E-05	5.1E-04	2.7E-06	2.1E-04	1.1E-06
Mn	3.9E+00	5.1E-01	3.1E-01	4.0E-02	1.1E-02	1.5E-03	9.8E-03	1.3E-03
Mo	3.6E-02	1.9E-04	3.2E-02	1.7E-04	6.0E-02	3.1E-04	4.1E-02	2.1E-04
Ni	3.6E-03	9.3E-05	1.9E-03	5.0E-05	4.0E-03	1.0E-04	2.9E-03	7.5E-05
Zn	2.7E-04	8.6E-07	3.3E-04	1.0E-06	2.1E-04	6.6E-07	1.7E-04	5.4E-07
HI[d]	4.1E+00	5.1E-01	7.0E-01	4.5E-02	7.2E-01	7.6E-03	6.0E-01	7.1E-03
Conditions	Aerobic condition				Anaerobic condition			
	With DWTR		Without DWTR		With DWTR		Without DWTR	
Element	HQ_{oral}	HQ_{dermal}	HQ_{oral}	HQ_{dermal}	HQ_{oral}	HQ_{dermal}	HQ_{oral}	HQ_{dermal}
Al	4.4E-04	2.3E-06	7.1E-05	3.7E-07	9.9E-04	5.1E-06	1.5E-03	7.7E-06
As	4.7E-01	2.6E-03	3.0E-01	1.7E-03	6.9E-01	3.8E-03	1.4E+00	7.5E-03
Ba	2.6E-02	1.9E-03	2.9E-02	2.1E-03	4.1E-02	3.1E-03	3.7E-02	2.8E-03

Cu	NC	NC	NC	NC	4.1E-04	2.1E-06	4.6E-04	2.4E-06
Fe	3.1E-04	1.6E-06	1.1E-04	5.9E-07	4.3E-01	2.2E-03	1.1E-01	5.7E-04
Mn	4.2E-03	5.5E-04	9.4E-03	1.2E-03	4.5E+00	5.8E-01	2.2E+00	2.8E-01
Mo	5.2E-02	2.7E-04	3.8E-02	2.0E-04	2.4E-02	1.2E-04	1.2E-02	6.0E-05
Ni	3.2E-03	8.2E-05	3.6E-03	9.3E-05	6.6E-03	1.7E-04	5.1E-03	1.3E-04
Zn	1.6E-05	4.9E-08	4.7E-05	1.5E-07	3.7E-03	1.1E-05	1.9E-03	6.0E-06
HI	6.0E-01	5.8E-03	4.4E-01	6.2E-03	5.7E+00	5.9E-01	3.8E+00	3.0E-01

[a] Oral hazard quotient;

[b] Dermal hazard quotient;

[c] Data cannot be calculated because the metal concentration in lake water was below detection limit;

[d] Hazard index for multiple substances and/or exposure pathways.

Table S7 Cancer risks for As in overlying lake water of sediments with and without DWTR.

Conditions	pH 5.5-6.0				pH 8.5-9.0			
	With DWTR		Without DWTR		With DWTR		Without DWTR	
Element	OCR[a]	DCR[b]	OCR	DCR	OCR	DCR	OCR	DCR
As	NC[c]	NC	9.9E-05	5.4E-07	2.5E-04	1.4E-06	2.1E-04	1.1E-06
Conditions	Aerobic condition				Anaerobic condition			
	With DWTR		Without DWTR		With DWTR		Without DWTR	
Element	OCR	DCR	OCR	DCR	OCR	DCR	OCR	DCR
As	2.1E-04	1.2E-06	1.4E-04	7.5E-07	3.1E-04	1.7E-06	6.2E-04	3.4E-06

[a] Oral cancer risk;

[b] Dermal caner risk;

[c] Data cannot be calculated because As concentration in lake water was below detection limit.

Water quality assessment based on comparison of metal/metalloid concentrations in lake water with water quality criteria

Metal/metalloid concentrations in lake water were compared with National Recommended Water Quality Criteria for fresh water in USA[16] and Environmental Quality Standard for Surface Water in China (GB3838-2002)[17]. The National Recommended Water Quality Criteria for fresh water in USA divided the metals/metalloids into two categories, i.e. priority pollutants, including Ag, As, Be, Cd, Cr, Cu, Hg, Ni, Pb, Sb, Se, and Zn, and non priority pollutants, including Al and Fe. The standards for the pollutants commonly had two thresholds, which were criteria maximum concentration (CMC) and criterion continuous concentration (CCC). The CMC is an estimate of the highest concentration of a material in surface water to which an aquatic community can be exposed briefly without resulting in an unacceptable effect. The CCC is an estimate of the highest concentration of a material in surface water to which an aquatic community can be exposed indefinitely without resulting in an unacceptable effect. Among the priority pollutants, Ag, Be, Cd, Co, Cr, Hg, Sb, Se, and Pb were not detectable in lake water, sediments or DWTR. The concentrations of As, Cu, Ni, and Zn in lake water were clearly below the CMC and CCC. For the non priority pollutants, the concentrations of Al were clearly below the CMC and CCC. However, Fe concentrations in lake water exceeded the CCC (Fe 1000 $\mu g\ L^{-1}$) under anaerobic condition, and DWTR addition increased the exceeding.

The Environmental Quality Standard for Surface Water in China (GB3838-2002)[17] divided surface water into five classes according to its purpose for use and protection target, and the standard of Class Ⅲ was adopted herein. The Class Ⅲ is mainly for class two protection areas for centralized potable water source, and protection areas for general fishing and swimming in China, and is referred to As, Cd, Cr, Cu, Hg, Se, Pb, and Zn. Besides the undetectable Cd, Cr, Hg, Se, and Pb, the concentrations of As, Cu, and Zn in lake water were clearly below the standards (Figure 2). There also have supplementary standards for the centralized potable water source, which include Fe and Mn. In comparison, the concentrations of Fe and Mn under anaerobic conditions and Mn under acidic (pH 5.5–6.0) condition remarkably exceeded the standards (Fe 300 μg L^{-1} and Mn 100 μg L^{-1}), and the exceeding increased by DWTR. In addition, the Ba and Mo were not included in the surface water (freshwater) standards used herein. However, the concentrations of Ba and Mo in lake water were clearly below the Standards for Drinking Water Quality in China (GB5748-2006: Ba 700 μg L^{-1} and Mo 70 μg L^{-1}). Therefore, it was considered that DWTR addition could not induce the Ba and Mo risks to environment.

The risk assessment code of metal/metalloid in sediments

Table S8 The calculated risk assessment code of the metals and As in sediments with and with DWTR addition.

Element	Sediments	Al	As	Ba	Be	Cd	Co	Cr	Cu	Fe	Mn	Mo	Ni	Pb	Zn
pH 5.5–6.0	With DWTR	N[a]	L[b]	H[c]	N	M[e]	M	N	N	L	VH[d]	N	L	N	L
	Without DWTR	N	L	H	N	M	M	N	N	L	VH	N	L	N	L
pH 8.5–9.0	With DWTR	N	L	H	N	M	M	N	N	L	VH	N	L	N	L
	Without DWTR	N	L	H	N	M	M	N	N	L	VH	N	L	N	L
Aerobic	With DWTR	N	L	H	N	M	M	N	N	L	VH	N	L	N	L
	Without DWTR	N	L	H	N	M	M	N	N	L	VH	N	L	N	L
Anaerobic	With DWTR	N	**N**	H	N	M	M	N	N	L	VH	N	L	N	L
	Without DWTR	N	**L**	H	N	M	M	N	N	L	VH	N	L	N	L

[a] No risk;

[b] Low risk;

[c] High risk;

[d] Very high risk;

[e] Medium risk.

Metal/metalloid contents in samples used in this study

Table S9 The total metal/metalloid contents in lake water, sediment, and drinking water treatment residue (DWTR), and the detection limits of ICP-AES.

Elements	Lake water (ug L^{-1})	Sediment[a] (ug g^{-1})	DWTR[b] (ug g^{-1})	Detection limits (ug L^{-1})
Ag	<DL[c]	<DL	<DL	46.71
Al	<DL	29000	107000	5.61
As	<DL	14	110	5
Ba	100	210	350	0.24
Be	0.60	1.1	0.42	0.06
Cd	<DL	0.60	0.56	0.21
Co	<DL	13	8.5	0.84
Cr	1.0	57	730	0.57
Cu	6.0	38	35	0.57
Fe	3.0	34000	110000	1.17
Hg	<DL	<DL	<DL	4.23
Mn	0.20	740	8600	0.12
Mo	4.0	2.0	9.2	1.23
Ni	<DL	41	23	0.6
Pb	<DL	29	12	2.97

Sb	<DL	<DL	<DL	3.54
Se	<DL	<DL	<DL	7.11
Zn	2.4	170	90	0.36

[a] The digestion ratio for sediment and DWTR is 1:100;

[b] The total metal/metalloid contents in DWTR were referred to Wang *et al.*[18];

[c] Below the detection limit.

The selected properties of overlying lake water in the incubation test

Table S10 The dissolved oxygen (DO), pH, and ORP of overlying water under different conditions.

pH	Time (d)	Sediments	DO (mg L^{-1})	Redox conditions	Time (d)	Sediments	pH	ORP (mV)	DO (mg L^{-1})
5.5–6.0	10	With DWTR	6.7±0.63[a]	Aerobic	10	With DWTR	7.8±0.11	-[b]	4.6±0.19
		Without DWTR	7.0±0.46			Without DWTR	7.8±0.07	-	5.6±0.11
	20	With DWTR	6.2±0.21		20	With DWTR	8.1±0.02	-	4.7±1.2
		Without DWTR	6.3±0.43			Without DWTR	8.3±0.04	-	6.3±0.07
	30	With DWTR	5.4±0.17		30	With DWTR	8.1±0.07	-	4.3±0.03
		Without DWTR	5.5±0.21			Without DWTR	8.1±0.03	-	5.1±0.29
8.5–9.0	10	With DWTR	5.0±0.24	Anaerobic	10	With DWTR	6.6±0.04	-222±1.3	-
		Without DWTR	6.2±0.49			Without DWTR	6.7±0.03	-246±1.5	-

20	With DWTR	5.0 ±0.30	20	With DWTR	6.7 ±0.10	-87 ±60	-
	Without DWTR	5.0 ±0.02		Without DWTR	6.7 ±0.02	-275 ±11	-
30	With DWTR	4.8 ±0.13	30	With DWTR	6.7 ±0.03	-308 ±33	-
	Without DWTR	5.2 ±0.02		Without DWTR	6.7 ±0.02	-232 ±4.6	-

[a] Mean ± standard deviation, n=2;

[b] Not measured.

The DO levels of lake water during the test of the pH effect were 4.8–7.0 mg L^{-1}, while during the test of the redox conditions effect, the DO levels of lake water under aerobic condition were 4.3–6.4 mg L^{-1}, and the oxidation-reduction potential of lake water under anaerobic condition was -87–-308 mV. In addition, the pH of lake water under anaerobic condition was 6.6–6.7, and under aerobic condition, it was 7.8–8.3.

The results of the metals and As fractionation in sediments

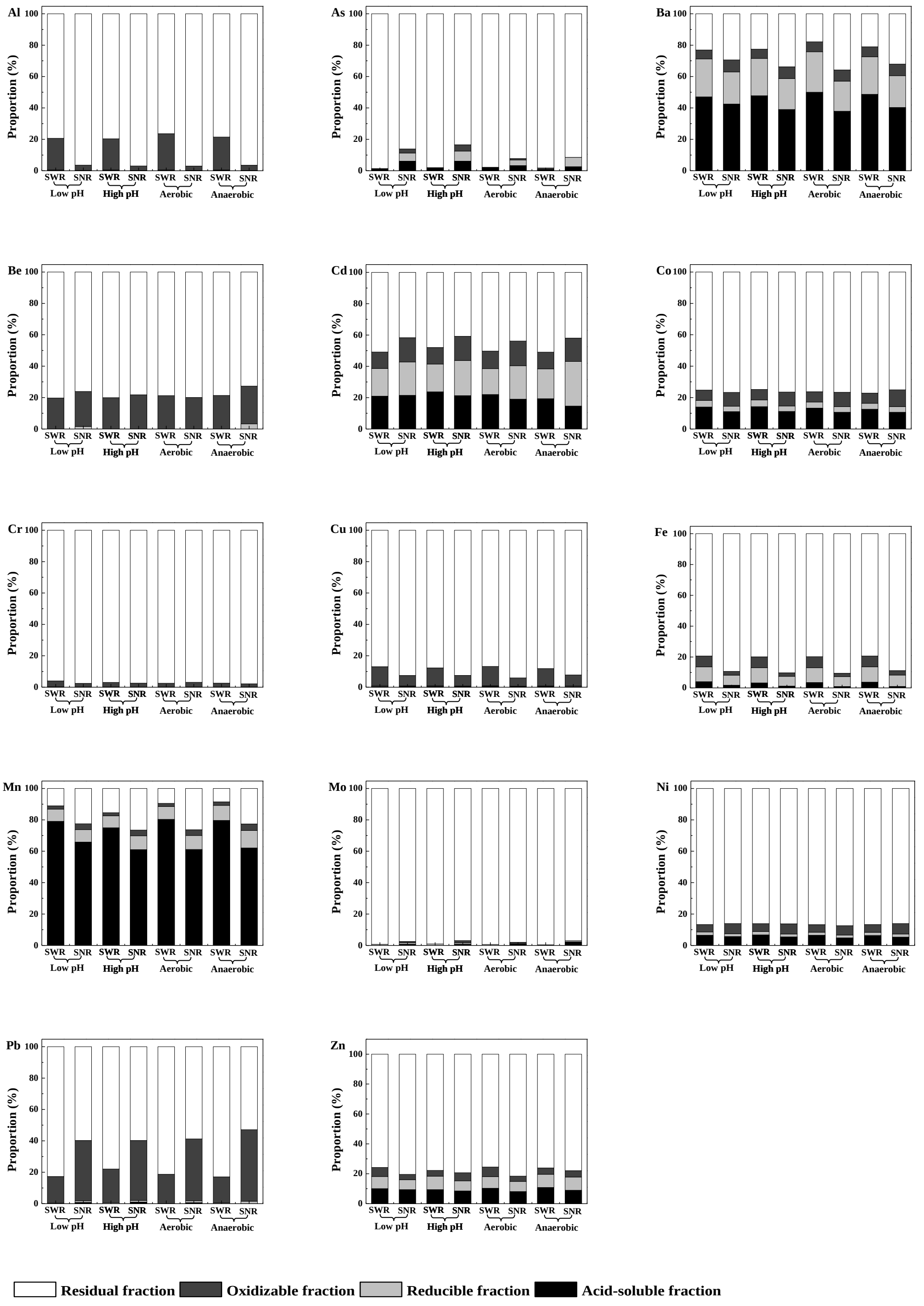

Figure S1. The results of the metals and As fractionation in sediments with and without DWTR addition after incubation tests. SWR and SNR represent sediments with and without DWTR, respectively; Low pH and High pH represent pH 5.5–6.0 and 8.5–9.0, respectively.

References

1. Wang, C.H., Qi, Y. & Pei, Y.S. Laboratory investigation of phosphorus immobilization in lake sediments using water treatment residuals. *Chem. Eng. J.* **209**, 379-385 (2012).
2. Yuan, C.G., Shi, J.B., He, B., Liu, J.F., Liang, L.N. & Jiang, G.B. Speciation of heavy metals in marine sediments from the East China Sea by ICP-MS with sequential extraction. *Environ. Int.* **30**, 769-783 (2004).
3. USEPA. *SW-846 method 3051: Microwave assisted acid digestion of sediments, sludges, soils and oils* (U.S. Environmental Protection Agency, 2007).
4. USEPA. *SW-846 Method 1311: Toxicity characteristic leaching procedure* (U.S. Environmental Protection Agency, 1992).
5. Wang, F., Bu, Q., Xia, X. & Shen, M. Contrasting effects of black carbon amendments on PAH bioaccumulation by *Chironomus plumosus* larvae in two distinct sediments: Role of water absorption and particle ingestion. *Environ. Pollut.* **159**, 1905-1913 (2011).
6. Xia, X., Chen, X., Zhao, X., Chen, H. & Shen, M. Effects of carbon nanotubes, chars, and ash on bioaccumulation of perfluorochemicals by *chironomus plumosus* larvae in sediment. Environ. Sci. Technol. **46**, 12467-12475 (2012).
7. Xue, P.Y. &Yan, C.Z. Arsenic accumulation and translocation in the submerged macrophyte *Hydrilla verticillata* (Lf) Royle. *Chemosphere* **85**, 1176-1181 (2011).

8. Menz, J., Schneider, M. & Kümmerer, K. Toxicity testing with luminescent bacteria – Characterization of an automated method for the combined assessment of acute and chronic effects. *Chemosphere* **93**, 990-996 (2013).

9. Ocampo-Duque, W., Sierra, J., Ferré-Huguet, N., Schuhmacher, M. & Domingo, J.L. Estimating the environmental impact of micro-pollutants in the low Ebro River (Spain): An approach based on screening toxicity with Vibrio fischeri. *Chemosphere* **72**, 715-721 (2008).

10. USEPA. *SW-846 chapter seven: Characteristics introduction and regulatory definitions, 4th edn* (U.S. Environmental Protection Agency, 2004).

11. Lim, H.S., Lee, J.S., Chon, H.T. & Sager, M. Heavy metal contamination and health risk assessment in the vicinity of the abandoned Songcheon Au–Ag mine in Korea. *J. Geochem. Explor.* **96**, 223-230 (2008).

12. USEPA. Risk assessment guidance for superfund volume I: Human health evaluation manual. Part A (interim final), EPA/540/1-89/002, Office of Emergency and Remedial Response, U.S. Environmental Protection Agency, Washington, D.C. (1989).

13. USEPA. *Risk assessment guidance for superfund volume I: Human health evaluation manual. Part E, supplemental guidance for dermal risk assessment (Final), EPA/540/R/99/005, Office of Superfund Remediation and Technology Innovation* (U.S. Environmental Protection Agency, 2004).

14. Hamidin, N., Yu, Q.J. & Connell, D.W. Human health risk assessment of chlorinated disinfection by-products in drinking water using a probabilistic approach. *Water Res.* **42**, 3263-3274 (2008).

15. USEPA, *Regional screening level (RSL) tapwater supporting table.* (2012) Available at: http://www.epa.gov/reg3hwmd/risk/human/rb-concentration_table/Generic_Tables/docs/restap_sl_table_01run_MAY2013.pdf

16. USEPA. *National recommended water quality criteria* https://www.epa.gov/wqc/national-recommended-water-quality-criteria-aquatic-life-criteria-table (2009).

17. EPA, C. *GB 3838–2002: Environmental quality standards for surface water (Chinese)* (China Environmental Science Press, 2003).

18. Wang, C.H., Yuan, N.N. & Pei, Y.S. Effect of pH on metal lability in drinking water treatment residuals. *J. Environ. Qual.* **43**, 389-397 (2014).
